# The Sarcoma Assessment Measure (SAM): Preliminary Psychometric Validation of a Novel Patient-Reported Outcome Measure

**DOI:** 10.3390/cancers16061096

**Published:** 2024-03-08

**Authors:** Lee Hulbert-Williams, Nicholas J. Hulbert-Williams, Ana Martins, Lesley Storey, Jennie Bradley, Hatty O’Sullivan, Lorna A. Fern, Maria Lawal, Rachael Windsor, Craig Gerrand, Jeremy S. Whelan, Lindsey Bennister, Mary Wells, Rachel M. Taylor

**Affiliations:** 1 Department of Psychology, Edge Hill University, Ormskirk L39 4QP, UK; 2 Cancer Clinical Trials Unit, University College London Hospitals NHS Foundation Trust, London NW1 2PG, UK; 3 Department of Psychology, Anglia Ruskin University, Chelmsford CM1 1SQ, UK; 4 Iqvia Ltd., Reading RG1 3JH, UK; 5 Paediatric Directorate, University College London Hospitals NHS Foundation Trust, London NW1 2PG, UK; 6 Sarcoma Unit, The Royal National Orthopaedic Hospital NHS Trust, Stanmore HA7 4LP, UK; 7 St Luke’s Hospice, Sheffield S2 1GQ, UK; 8 Nursing Directorate, Imperial College Healthcare NHS Trust, London W2 1NY, UK; 9 Department of Surgery and Cancer, Imperial College London, London SW7 5NH, UK; 10 Centre for Nurse, Midwife and Allied Health Profession Led Research (CNMAR), University College London Hospitals NHS Foundation Trust, London NW1 2PG, UK; rtaylor13@nhs.net; 11 Department of Targeted Intervention, University College London, London NW1 2PG, UK

**Keywords:** soft tissue sarcoma, bone tumours, gastrointestinal stromal tumours, quality of life, patient-reported outcome

## Abstract

**Simple Summary:**

The Sarcoma Assessment Measure (SAM) is a special questionnaire for patients with sarcomas, a type of cancer. It was created with input from both patients and healthcare professionals and is meant to be used by professionals to better understand how sarcoma affects a patient’s life. We tested the SAM on 762 patients who had different types of sarcomas and ranged in age from 13 to 82. We found that the SAM could be a useful tool for both researchers and healthcare professionals to assess how sarcoma symptoms impact a patient’s life. However, more testing with a larger and more diverse group of patients is needed to be sure it is a good outcome measure in drug trials.

**Abstract:**

The Sarcoma Assessment Measure (SAM) was developed as a sarcoma-specific patient-reported outcome measure to be used in clinical practice. We have reported in detail how SAM has been developed in collaboration with patients and healthcare professionals. The aim of this paper is to report the preliminary validation of SAM. The 22-item SAM was administered alongside a validated quality of life questionnaire and measure of activities of daily living. Linear modelling was used to build a measure, which had predictive validity in comparison to more established outcome measures. Of the 762 patients who participated in the study, 44.1% identified as male, and participant age ranged from 13 to 82 years. Clinically, participants presented with a range of soft tissue (82.2%) and bone (21.8%) sarcomas. Our preliminary analysis indicates that SAM accounts for 35% of the global quality of life scale and 18% of the Toronto Extremity Salvage Scale (TESS); so psychometrically, it overlaps with quality of life and activities of daily living, but also measures distinct concerns. This demonstrates that this measure picks up issues that are important to patients with sarcoma that are not reflected in other measures. We have established the preliminary validity of SAM and believe it has utility as a patient-reported outcome measure both as a research tool and for assessing the impact of symptoms and dysfunction related to sarcoma as part of clinical care. Further validation using a larger and more clinically diverse sample is now needed.

## 1. Introduction

Sarcoma is a heterogeneous group of cancers of the connective tissue, which are inclusive of over 100 subtypes presenting as soft tissue, bone tumours, and gastrointestinal stromal tumours (GIST). Tumours can therefore be present in any part of the body, with the majority presenting in the upper and lower extremities. The treatment burden for sarcoma is high, often requiring extensive surgery, high-dose chemotherapy, and radiotherapy. Sarcomas are also associated with a high risk of developing metastases, recurrence, and poorer survival in comparison to the common cancers [1,2,3]. Further heterogeneity exists among patients with sarcoma because they can present from birth to old adulthood, and some subtypes are a common cancer in younger people [4].

The high risk of developing metastases and recurrence has resulted in treatment pathways in many countries, including the United Kingdom (UK), to comprise annual follow-up often accompanied with a scan. Repeated hospital attendances have a significant impact on patients’ emotional well-being [5], as these require patients to revisit the experiences they had at diagnosis [6]. For this and other reasons, fears of sarcoma recurrence are common [7]. Clinical consultations are driven by these emotions, so patients either focus on what their treatment options are if the scans are positive or experience relief if they are clear. This relief overwhelms everything, so patients often forget anything additional they want to discuss with their clinician [8,9]. One way to overcome this is using outcome measures within clinical practice to help direct meaningful communication between clinicians and patients. However, to be meaningful, the outcome measure needs to reflect issues that are important to the patient and are representative of the disease.

Documenting the patient-reported experience of healthcare and outcomes is now considered a central component in providing and evaluating quality cancer care in the UK [10]. Aligned to the importance of patient-reported outcomes (PRO) has been the increase in instruments to measure them. This has progressed from generic measures that can be used across all disease states, such as the Short-Form-36 (SF36) [11], to condition-specific versions, for example, the European Organisation for Research and Treatment in Cancer Quality of Life Questionnaire (EORTC QLQ-C30) [12]. Adoption of patient-reported outcome measures (PROM) into clinical practice can improve health-related processes, outcomes, and satisfaction with care; however, significant barriers to adoption and implementation are often reported, including length and perceived burden on the clinical teams [13], fear of bringing up topics of conversation that clinicians are not trained or equipped to follow through on [14], or that more generalisable measures may not be perceived as being patient-centred enough to be clinically useful [15].

The importance of using condition- or disease-specific measures also follows the observation that generic measures are often not responsive to small changes in clinical condition and functioning, and they may overlook clinically relevant aspects related to the specific condition [16]. Interpretation of results from generic measures may also be challenging in discerning assessment of overall health in relation to the patient’s specific condition. Conversely, disease-specific measures may not be comprehensive enough to allow comparison to other conditions, where the normative data from generic measures enable interpretation to be more meaningful [16,17,18]. Administering PROMs alongside condition-specific measures is, therefore, recommended to capture the burden of disease whilst also facilitating comparisons to other populations.

Relating specifically to the age distribution of those diagnosed with sarcoma, very few validated PROMs span all age ranges and accommodate variance relating to developmental stages. Validation in specific target populations is critical; however, it is important to ensure that PROMs can be appropriately used in clinical practice and are applicable for assessing population-specific challenges over longer periods of follow-up and surveillance.

While many generic and generic-cancer PROMs often provide an additional cancer-type module (see EORTC: https://qol.eortc.org/ (accessed on 14 January 24); FACIT: https://www.facit.org/ (accessed on 14 January 24); PROMIS: https://www.promishealth.org/ (accessed on 14 January 24)), there are currently no PROMs specific for sarcoma. Measures currently available for sarcoma include the BtDux, which is specifically for teenagers and young adults with bone tumours [19]; the Soft Tissue Sarcoma questionnaire, which specifically focuses on symptoms associated with six soft tissue subtypes [20]; the Toronto Extremity Sarcoma Scale (TESS), which measures the impact of upper and lower limb sarcoma on activities of daily living [21]; and the Gounder/DTRF Desmoid Symptom/Impact Scale (GODDESS), which has been developed for patients with desmoid tumours or aggressive fibromatosis [22].

Due to the heterogeneity of sarcoma, there have been arguments that one outcome measure could not adequately reflect the impact of all subtypes [23]. There is also a clinical perception that patient-reported outcome, especially quality of life, is influenced by sarcoma-related features, such as tumour site and subtype or type of treatment. However, evidence to date does not support differences according to type of surgery [24] or tumour location [25]. Poorer quality of life may be seen according to age; however, in adolescents and young adults reported as having poorer quality of life than older adults and the elderly [26], generic consequences of cancer treatment per se, especially pain and fatigue, have been noted to have a greater association with poorer quality of life [27,28]. The lack of specificity of existing PROMs have led to repeated calls for measures to be developed specifically for patients with sarcoma [24,25,26,29].

To address this existing gap in available PROMs for sarcoma, we aimed to develop and validate an outcome measure that better reflects the patient experience of living with a sarcoma diagnosis across the lifespan. The PROM was based on the following definition of health-related quality of life: “… subjective, multidimensional, and dynamic. It is unique to each individual and includes aspects of physical, psychological and social function. It is dependent upon not only the stage of development but also the illness trajectory. This involves the achievement of goals and aspirations and the constraints imposed through ill health and treatment” [30].

**Figure 1:**
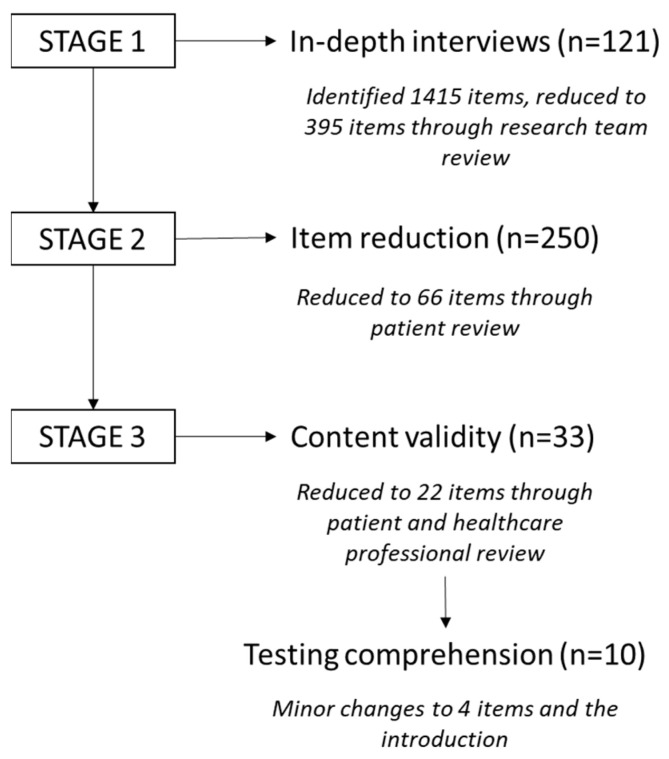
Summary of the development of the Sarcoma Assessment Measure (SAM) [32].

The study used the broad methodology employed for developing quality of life measures in cancer [31]; significantly, content selection decisions were driven by patient experience rather than researcher or clinician bias to remain true to the ”subjective” aspect of the above definition. Previous reports on the development of our novel Sarcoma Assessment Measure (SAM) are presented in detail elsewhere [8,9,32], and the methods of development are presented in Figure 1 [24,32].

The SAM was developed to be a measure that could guide clinical consultations and therefore focused on the issues that were more important and impacting on patients’ outcome. This phase of the study aimed to validate the SAM in a larger sample in order to derive an appropriate scoring method and assess suitability for use of the SAM in clinical care.

## 2. Materials and Methods

### 2.1. Study Design

This was a prospective cross-sectional survey, recruiting patients from across the United Kingdom (UK). The study was approved by the London–Riverside Research Ethics Committee (ref: 18/LO/0023) and survey administration was coordinated by Quality Health (IQVIA Ltd., Reading, UK).

### 2.2. Sample and Setting

Patients were eligible to participate if they had a diagnosis of sarcoma, were aged 13 or older, and were able to communicate verbally or in written English. In line with general recommendations for PROM development (e.g., Brysbaert [33]) our aim was to recruit a minimum of 220 participants (10 participants per item in SAM). However, due to the heterogeneous nature of sarcoma, a larger sample was sought in order that variance according to clinical factors could be explored [9].

Patients were recruited through three mechanisms. First, we recruited directly through participating hospitals. For young people aged 13–15 years, the study was explained to the parent/guardian, and if they were happy for their child to participate, it was explained to the young person, who then received the questionnaire if they too expressed interest. Second, we recruited through the National Cancer Patient Experience Survey (NCPES), which was coordinated by Quality Health, the original contracted hosts of the NCPES. When patients returned this survey, they had the opportunity to leave contact details to be invited to participate in future research. Patients participating in the 2014–2017 NCPES with a diagnosis of sarcoma who provided future research consent were approached for this study. Finally, we re-invited those who had participated in the development phases of the study [32]. Details of these participants were transferred securely to Quality Health. Regardless of identification method, all patients were given or sent information, and the return of the questionnaire was implicit of consent. SAM was approved by the London–Riverside Research Ethics Committee (reference 18/LO/0023), the Health Research Authority, and the Research and Development department in each participating hospital.

### 2.3. Data Collection

Data to test the psychometric properties of the SAM were collected using established, validated questionnaires. These were administered as a single questionnaire pack in paper format sent postally by Quality Health or given directly to patients in participating hospitals. Patients were instructed to complete the questionnaires without help and leave anything blank that was unclear. These were returned in a pre-paid envelope. No reminders were sent to patients recruited through participating hospitals. Patients sent questionnaires by Quality Health were sent two reminders after 2 and 4 weeks, and the data collection process was entirely managed by Quality Health.

The version of the SAM used in this study (version 1.0) contained 22 items, each scored on a 5-point Likert-type scale ranging from “strongly agree” to “strongly disagree”, with the option of responding as “not applicable” (see tables in results section for included items). The purpose of this study was to establish whether any items were redundant and to establish a method of scoring the SAM.

Alongside our novel SAM, we included two additional questionnaires with established reliability and validity in cancer patients. These were selected due to the frequency with which they have previously been used in other studies involving patients with sarcoma [24]. First, we included the EORTC QLQ-C30 [12], a 30-item measure of quality of life, incorporating 9 multiple-item scales: 5 functional scales (physical, role, emotional, social, and cognitive); 3 symptom scales (fatigue, pain, and nausea and vomiting) and a global health and quality of life scale. Five single items assess the physical symptoms of dyspnoea, insomnia, appetite, diarrhoea, and constipation, and 1 item evaluated the financial impact of the disease. The first 28 items on the questionnaire are rated on a response scale from 1 (“not at all”) to 4 (“very much”), and compose the functioning, symptom, and financial difficulties scales. Items 29 and 30, which assess global health/QOL, use a response scale ranging from 1 (“very poor”) to 7 (“excellent”). Scores on the EORTC QLQ-C30 are transformed to a 0–100-point scale. Higher mean scores for the functional scales and global health status/QOL scale represent better functioning and overall QOL. While the QLQ-C30 is demonstrably reliable and valid for patients over 18 years, it has also been used in younger adolescents [34]. Higher mean values for the multi-item symptom scales and higher scores for single items represent more frequent and/or more intense symptoms and a higher financial impact.

Second, we included the TESS. There are two versions of the TESS to reflect upper and lower extremity limitations in daily life, such as restrictions in body movement, mobility, self-care, and performance of daily tasks and routine [21]. These are commonly used in clinical practice in patients with extremity sarcoma. Items reflect activities of daily living that could be impacted by upper/lower limb disability rather than treatment side-effects. With permission from the author (personal correspondence), the upper and lower extremity versions were combined so it could be administered as a single measure. It included 17 unique upper and 16 lower extremity items, and 13 that were common across both (total of 46 items). Patients can answer questions concerning activities they do not perform in daily life with “not applicable.” The degree of physical disability is rated from 0 (not possible) to 5 (without any problem). Higher total scores indicate fewer functional limitations. The two final items, which related to overall perceptions of activity and disability, were left as stand-alone items.

### 2.4. Analysis

Neither traditional factor analytic approaches using the classical test theory (CTT) nor the alternative item response theory (IRT) approaches were suitable for this kind of checklist measure. Both CTT and IRT approaches assume that within a potential new measure, items will group together because they are each assessing a different aspect of a coherent underlying psychological construct; for example, the Hospital Anxiety and Depression Scale (HADS; Zigmond and Snaith [35]) is suitable for these analyses because a sub-set of items collectively assess anxiety as a construct, and the remainder assess depression. Here, we had developed a list of items that could be best described as a problem checklist: not all participants were expected to identify with the relevance of all items (e.g., not everyone will be using a prosthesis or taking painkillers). Though the items on the checklist might all be caused by sarcoma, there is substantial variation in the presentation of sarcoma such that the measure would not meet the strict assumption of causation by a latent variable which underlies CTT and IRT (see Loehlin and Beaujean [36] for a discussion).

An alternative approach might have been to ask respondents to weight each item on the perception of importance, as has been done with psychometric scales such as the Social Readjustment Rating Scale [37]. However, Cox et al. [38] warn against such approaches, as subjective weighting techniques can be erratic and lead to implausible conclusions. As Cox et al. (p. 34) conclude: “Given the general lack of a pre-eminent weighting scheme, a prudent course includes checks on the robustness of conclusions to alternative, but still arbitrary, choices of weighting schemes”.

Consequently, to test whether a scoring scheme might be desirable in principle, we adopted a pragmatic approach to calculating a total score. First, we undertook a separate regression analysis for each item of the SAM to calculate how much each one predicted global QOL as measured by the EORTC QLQ-C30 (our measure of convergent validity). To minimise respondent burden, it is generally desirable to remove items which contribute poorly to convergent validity, and so we removed the three items which predicted 0% of the variance in global QOL (rounded to two decimal places). We then compared two approaches of calculating an overall score: first, a simple mean score from all items; and second, a weighted mean with weights determined by their regression coefficient of prediction of global quality of life. We used an odd-even data split, creating Subsamples A and B, to avoid over-fitting the data: the first sample (odd numbered respondents) was used to create scoring weights using the regression analysis, and the second sample (even numbered respondents) was used to calculate total scores on which to test comparative efficacy of the two approaches. Given the level of ”not applicable” responses, we adopted a generous cut-off for pro-rating in calculating total scores: where 10 or more items had been completed, a mean score was generated from the questions they did answer. Higher scores represent better functioning.

## 3. Results

Valid questionnaires were received from a total of 762 participants (Table 1). The majority of patients had soft tissue sarcoma (72%) and were off treatment (77%). Despite our best efforts to recruit a younger sample, the mean age was 63 years (SD = 17).

The mean score on the TESS was 180 (SD = 52; N = 614). Of the total sample, 68 (9.1%) rated their participation in activities of daily living as being “extremely difficult” or “impossible” to do, and 34 (4.5%) stated that they were either “severely disabled” or “completely disabled”. For this paper, we used only the QLQ-C30 global quality of life score; within this sample, we recorded a mean score of 70.3 (SD = 20.6).

### 3.1. Acceptability of the SAM

Table 2 summarises the number of respondents and the median and inter-quartile range (IQR) of the scores for each item. Sixteen questions were responded to by more than 75% participants. The six questions with a lower response related to items targeted at specific groups: amputations (questions 5 and 6), extremity surgery (question 4), younger patients (questions 8 and 19), and those experiencing pain (question 7).

### 3.2. Scoring the SAM

Table 3 summarises results from the regression coefficient models in which we used each item of the SAM to predict global QOL. Based on these data, three items predicted <2% of the variance (number 2, 11, and 15), so these were removed from further analysis.

Pearson’s r correlation tests were then used to compare correlations between the weighted and unweighted mean scores with both global quality of life and TESS total scores (see Table 4). The SAM weighted mean score explained 1.7% more variance in total TESS scores and approximately 1.2% extra variance in global QOL than the simple unweighted mean. The SAM therefore explained 34.8% of the variance in global QOL and 18.5% of TESS.

## 4. Discussion

The purpose of this study was to develop an outcome measure that could be used to guide clinical consultations. We also wanted to be able to derive a scoring formula to enable patients and/or clinicians to monitor changes over time. Health models suggest clinical care only contributes to 20% of clinical outcome [39], and therefore, we wanted to base the content of the measure on the aspects of living with a sarcoma diagnosis that were most important to patients. While some authors equate “experience” to “experience of healthcare” [23]—the biomedical model of assessing outcome—we aimed to take a more holistic person-centred approach. Patients are more than their illness, and after a sarcoma diagnosis, we strive to support patients to reintegrate to their pre-diagnosis lives or to find their “new normal” [8,9]. Having an outcome measure that goes beyond symptoms is therefore important. Whilst symptom-based items were included in SAM, (e.g., pain), the measure assessed the personal impact of that symptom, for example, whether it was manageable using pain-relieving medications.

The conceptual basis for the SAM was a model of quality of life [30], so it included items related to the three domains of health (physical, emotional, and social functioning) [40]. However, it was interesting to note in the development phase of SAM, there was a preponderance of items rated highly important and impactful by patients within the emotional well-being domain [32]. This concurs with previous literature demonstrating a prevalence of anxiety and depression in patients with sarcoma in a fifth to a third of patients [41,42]. We have also identified fear of recurrence as having a significant impact on patients’ lives [5], which was higher than reports in other types of cancer [7]. Given the seemingly higher weighting patients place on the emotional impact of sarcoma, and the relatively superficial exploration of this in the literature, this is an area that warrants more detailed investigation.

Based on our regression analyses, we excluded three of the twenty-two items from further analysis (2. I am more conscious of what I eat since I was diagnosed with sarcoma; 11. Since my diagnosis I appreciate everyday things more; 15. I try and cope emotionally on my own). Given our sample size, we are confident that these items do not contribute to our knowledge of the impact of sarcoma for most participants and so can be excluded from future iterations of the SAM.

Our results show that a weighted mean score on the SAM explained slightly more variance in TESS total scores than a simple mean, suggesting that not all questions are equally important in predicting outcomes. It is likely that for maximised predictive validity, future users of the SAM may want to apply a weighting in calculating total scores. Whilst we provide the regression weights from our sample, researchers and clinicians will need to decide whether our sample is sufficiently similar to their own participants and clients in deciding whether to use these weights or to re-weight the items based on fresh data. It is also worth noting that the increased validity lent to SAM by using a more complex scoring approach is apparently minimal, so users may prefer to calculate a more parsimonious unweighted total score.

During the development of SAM, there appeared to be increased awareness across the sarcoma community of patient-reported outcomes and an interest in using SAM, not just for clinical practice, but also as an endpoint in clinical trials and biomarker studies, e.g., ICONiC (Improving Outcome Through Collaboration in Osteosarcoma). The lack of sensitivity and/or specificity of existing generic cancer PROMs [24] has highlighted the challenge of including these in research in sarcoma if there is the potential no difference will be detected over time. Consequently, there have been several subtype-specific PROMs developed for leiomyosarcoma, synovial sarcoma, liposarcoma, or undifferentiated [20] and desmoid tumours or aggressive fibromatosis [22]. These are undoubtably valuable during the treatment phase of the patient journey, but we found a significant flooring effect with the EORTC QLQ-C30 symptom scales; given most of our participants were off treatment, this suggests that these were not symptoms experienced by this broader population of cancer survivors. As patients with sarcoma were not included in the development of the original EORTC measure and given that it was developed over 30 years ago when treatment for sarcoma was quite different, this is not surprising. It also raises the question whether a PROM developed from a biomedical perspective or focused more on the physical aspects of health will be suitable as an outcome in complex intervention trials, for example, those testing psychological and behavioural interventions.

We are confident that SAM has clinical relevance, especially as clinicians and patients were involved in every stage of the development and testing. However, we are cautious about the use of the current version as a sole outcome measure in biomedical studies, as this is beyond the reason for its development. We are currently making changes to the wording and structure of SAM, for example, adding filter questions for those items targeted at specific populations, e.g., amputees, childbearing age. We are now revalidating SAM-2 with these additional changes to be a PROM that can be used in research.

### Limitations

The current study had several limitations. First, although we aimed to capture a broad range of participants through multiple recruitment methods, we had under-representation of patients with GIST. The number of adolescents and young adults participating was also small (11%), so whilst the SAM as presented here might reflect the sarcoma-related experience overall, it may miss the nuances of developing sarcoma at this earlier life stage, which is recognised as being a challenging time to be diagnosed with cancer [43,44,45]. Further validation in these groups may therefore be required. Second, we struggled with the amount of missing data in this dataset because of the response options provided to participants. It is obvious from the list of items that not all items in SAM will be applicable to all sarcoma patients. For example, not all will be taking painkillers (item 7) or living with a prosthesis (items 5 and 6). Closer analysis of these items shows that although a substantial number of participants responded, “not applicable”, a large proportion also missed these items out entirely. We can assume here that some participants were leaving the question blank because it was irrelevant to them. A consequence of this is that we cannot be certain that data in SAM were missing not at random, which complicates how we can interpret and deal with missing data [46]. It will be necessary in developing the next version of SAM to alter item response options. One way to do this might be to replace statements of agreement with statements of scale of the problem (i.e., instead of strongly disagree to strongly agree, we could use definitely not a problem for me to definitely a problem for me). Alternatively, it might be possible to present the “not applicable” option in a way that makes the explicit meaning clearer; for example, for item 5 (“my prosthesis is heavy and uncomfortable”) the “not applicable” option could be clarified as “I don’t wear a prosthesis”, or for Item 7 it could be clarified as “I don’t take painkillers”. Finally, while we recruited a large number of participants, we had no mechanism to track the number of patients who were approached, and therefore we do not know the response rate. This would be helpful as an indicator of importance to patients but would also provide an estimate for future studies using SAM. Despite these limitations, this study recruited a substantial number of people who had been diagnosed with a range of sarcoma types, from across the UK, and represents the first PROM for patients with all types of sarcomas. Moreover, we were able to demonstrate the broad applicability and acceptability of a PROM designed to capture the holistic experience of those living both with and beyond treatment for sarcoma.

## 5. Conclusions

Our study aimed to validate a PROM for patients aged 13 onward with sarcoma. We are confident that SAM provides a comprehensive patient-reported outcome of multiple domains of the impact of sarcoma and its treatment, and that it is possible to generate a meaningful score of the total impact for individual patients. Given correlations with both global quality of life and total TESS scores, the SAM has high clinical relevance and could be a useful adjunct to clinical consultation discussions. Importantly, we have demonstrated that SAM picks up issues that are important to patients with sarcoma that are not reflected in other measures. With some minor alterations to response options, our data indicate that SAM is likely a useful outcome measure for treatment trials and patient experience research in this population.

## Figures and Tables

**Table 1:** Characteristics of participants.

Characteristic		Number	%
Gender	Male	349	46
	Female	407	54
	Unknown	6	
Marital status	Married/civil partnership/long-term relationship	479	64
	Widowed	88	12
	Single	71	9.5
	Cohabiting	59	7.9
	Divorced	50	6.7
	Unknown	15	
Sexuality	Heterosexual/Straight	669	96
	Prefer not to say	13	1.9
	Gay or Lesbian	9	1.3
	Bisexual	6	0.9
	Unknown	65	
Gender identity	Cisgender	685	99.7
	Transgender	2	0.3
	Unknown	75	
Age	13–39	84	11
	40–64	231	31
	65+	434	58
	Unknown	14	
Ethnicity	White	714	95
	Asian/Asian British	17	2.3
	Black/African/Caribbean/Black British	11	1.5
	Mixed	5	0.7
	Other	2	0.3
	Unknown	13	
Employment status	Employed	213	28
	Retired	380	52
	In education/training	15	2
	Other	129	17
	Unknown	27	
Type of sarcoma	Soft tissue	549	72
	Bone	166	22
	GIST	78	10
	Multiple	46	6
Site of tumour	Lower limb	209	27
	Abdominal organ	143	19
	Head and neck	133	17
	Pelvis	76	10
	Upper limb	74	9.7
	Chest	57	7.5
	Spine	25	3.3
	Other	186	24
Amputation	No	614	84
	Not applicable (did not have surgery)	63	8.6
	Yes	54	7.4
	Unknown	31	
Treatment	Surgery alone	274	37
	Surgery and radiotherapy	177	24
	Chemotherapy and surgery	121	17
	Surgery, radiotherapy and chemotherapy	65	8.9
	Chemotherapy alone	39	5.3
	Radiotherapy and chemotherapy	20	2.7
	Radiotherapy alone	16	2.2
	No treatment	12	1.6
	None of these combinations ^1^	9	1.2
	Unknown	29	
Current status	On treatment	169	23
	Being followed up only	551	77
	Unknown	42	

GIST: gastrointestinal stromal tumour. ^1^ A treatment combination not specified above.

**Table 2:** Acceptability of the items in the SAM, including the number of missing/not applicable responses.

Item	N		IQR		Missing or N/A
		Median	Lower	Upper	
1. I do whatever I can to keep healthy	753	4	4	5	9
2. I am more conscious of what I eat since I was diagnosed with sarcoma	728	4	3	4	34
3. I can do everything without help	735	4	2	5	27
4. My arm/leg is not as strong as it was before diagnosis	482	4	3	5	280
5. My prosthesis is heavy and uncomfortable	109	3	2	4	653
6. My prosthesis fits well enough to do the things I want to	104	4	2.75	4	658
7. My painkillers don’t take all the pain away	264	4	3	4	498
8. I worry about whether I will be able to have a family	118	3	1	4	644
9. I worry that my sarcoma may return	666	4	3	5	96
10. I feel anxious before my scan/appointment	697	4	3	4	65
11. Since my diagnosis I appreciate everyday things more	735	4	4	5	27
12. I have not accepted how sarcoma has changed my body	658	2	2	3	104
13. I try to keep a sense of humour	740	5	4	5	22
14. I focus on what I can do rather than what I can’t do	696	4	4	5	66
15. I try and cope emotionally on my own	707	4	4	5	55
16. I put fears about my sarcoma to the back of my mind	727	4	3	4	35
17. I have friends/family I talk to about things I worry about	728	4	4	5	34
18. I am self-conscious of my physical appearance	604	3	2	4	158
19. I have been able to go back to work/university/school	327	4	2	5	435
20. My friends/family treat me normally	719	5	4	5	43
21. I find the costs of travelling to and from the hospital difficult to meet	573	2	2	3	189
22. My treatment for sarcoma has affected my intimacy with others	610	3	2	4	152

**Table 3:** Regression models predicting global quality of life from each SAM item (using Subsample A).

Item	Beta	Std Error	*t*	*p*	Adj. R^2^
1. I do whatever I can to keep healthy	7.06	1.51	4.69	<0.001	0.05
2. I am more conscious of what I eat since I was diagnosed with sarcoma	0.04	1.04	0.04	0.969	0
3. I can do everything without help	7.99	0.68	11.8	<0.001	0.28
4. My arm/leg is not as strong as it was before diagnosis	−2.88	1.02	−2.82	0.005	0.03
5. My prosthesis is heavy and uncomfortable	−7.99	2.05	−3.9	<0.001	0.25
6. My prosthesis fits well enough to do the things I want to	7.66	2.54	3.02	0.004	0.17
7. My painkillers don’t take all the pain away	−4.6	1.54	−2.98	0.003	0.06
8. I worry about whether I will be able to have a family	−3.34	1.76	−1.89	0.063	0.04
9. I worry that my sarcoma may return	−4.7	1.13	−4.15	<0.001	0.05
10. I feel anxious before my scan/appointment	−2.27	0.94	−2.41	0.017	0.01
11. Since my diagnosis I appreciate everyday things more	0.01	1.25	0.01	0.992	0
12. I have not accepted how sarcoma has changed my body	−5.92	0.93	−6.36	<0.001	0.11
13. I try to keep a sense of humour	7.13	1.74	4.09	<0.001	0.04
14. I focus on what I can do rather than what I can’t do	5.6	1.47	3.82	<0.001	0.04
15. I try and cope emotionally on my own	1.39	1.11	1.25	0.212	0
16. I put fears about my sarcoma to the back of my mind	4.17	1.03	4.05	<0.001	0.04
17. I have friends/family I talk to about things I worry about	4.2	1.18	3.56	<0.001	0.03
18. I am self-conscious of my physical appearance	−4.87	0.87	−5.62	<0.001	0.09
19. I have been able to go back to work/university/school	6.49	1.04	6.23	<0.001	0.2
20. My friends/family treat me normally	8.78	1.38	6.38	<0.001	0.1
21. I find the costs of travelling to and from the hospital difficult to meet	−3.24	0.98	−3.31	0.001	0.03
22. My treatment for sarcoma has affected my intimacy with others	−5.59	0.88	−6.34	<0.001	0.12

**Table 4:** Correlation coefficients of both scored versions of the SAM against construct validity measures (using Subsample B).

**Parameter 1**	**Parameter 2**	**r**	**CI**	**t**	***p***	**Method**	**N**
C30 global QOL	SAM total prorated	0.58	[0.51, 0.65]	13.14	<0.001	Pearson	338
C30 global QOL	SAM total weight prorated	0.59	[0.51, 0.65]	13.24	<0.001	Pearson	338
TESS total	SAM total prorated	0.41	[0.31, 0.51]	7.58	<0.001	Pearson	279
TESS total	SAM total weight prorated	0.43	[0.33, 0.52]	7.93	<0.001	Pearson	279

TESS: Toronto Extremity Salvage Score; QOL: quality of life.

## Data Availability

Data are available on request from the authors.
